# The Nature of Job Crafting: Positive and Negative Relations with Job Satisfaction and Work-Family Conflict

**DOI:** 10.3390/ijerph16071176

**Published:** 2019-04-02

**Authors:** Margherita Zito, Lara Colombo, Laura Borgogni, Antonino Callea, Roberto Cenciotti, Emanuela Ingusci, Claudio Giovanni Cortese

**Affiliations:** 1 Department of Business, Law, Economics and Consumer Behaviour “Carlo A. Ricciardi”, Università IULM (International University of Languages and Media), 20143 Milan, Italy; margherita.zito@iulm.it; 2 Department of Psychology, University of Turin, 10124 Turin, Italy; claudio.cortese@unito.it; 3 Department of Psychology, La Sapienza University, 00185 Rome, Italy; laura.borgogni@uniroma1.it (L.B.); roberto.cenciotti@uniroma1.it (R.C.); 4 Department of Human Sciences, Libera Università Maria SS. Assunta (LUMSA) University, 00193 Rome, Italy; toninocallea@libero.it; 5 Department of History, Society and Human Studies, University of Salento, 73100 Lecce, Italy; emanuela.ingusci@unisalento.it

**Keywords:** job crafting, work-family conflict, job satisfaction, job autonomy, JD-R model, structural equations model

## Abstract

This study investigated job crafting as mediator and its relation with job satisfaction and work-family conflict, considering job autonomy as antecedent. The research involved 389 participants working in a public organization. A structural equations model was estimated revealing that job autonomy is positively associated with job crafting and job satisfaction, and negatively associated with work-family conflict. Job crafting is positively related with job satisfaction and work-family conflict, as adverse effect of job crafting. As regards mediated effects, results show positive associations between job autonomy and both job satisfaction and work-family conflict through job crafting. This study contributes to literature, considering positive and negative outcomes, covering the lacking literature on job crafting and work-family conflict, and suggesting implications for employees’ well-being.

## 1. Introduction

In the last decades, different changes have characterized the working domain. In particular, the global crisis led organizations to be more competitive, to develop innovations and knowledge, and to produce and use new technology [1], which makes organizations more appealing and sustainable, and people more employable, since they work developing skills and abilities [2]. Because employees are asked to update their skills regarding innovations and to work in a more flexible way, an important process that involves a new job design is needed, also to face in a proactive way these changes [2]. This process is called job crafting, and it allows employees to model their own job by changing physical and cognitive—even relational—aspects of the working activity [3]. Job crafting is considered a form of proactive behaviour, since employees can change different job elements to adapt them to their needs, skills and preferences [4], improving their working conditions [5]. Considered the many changes in the work contexts, and the importance of the active role of employee in managing general working changes, another point to consider is the change in the workforce characteristics. The labour market has seen an increase of the proportion of women, even more educated, and also an increment of the older population [5,6], leading to a redefinition of family management. Therefore, job crafting can be used not only to deal with the new job system, but also to face the needs of the new workforce [5], including the needs for work-family balance. In fact, such a new work situation has blurred the boundaries between work and family, increasing the level of integration and decreasing the level of segmentation between work and family [7]. Job crafting could be a practice to achieve well-being since employees can shape their job while managing personal needs [8].

The aim of this study is to detect the mediating role of job crafting to understand its relation with job autonomy as antecedent, and with two outcomes: one positive, job satisfaction, and one negative, work-family conflict. This study contributes significantly to the job crafting literature, since traditionally job crafting is detected with positive outcomes, whereas this paper considers also a negative outcome. It is important to understand the possible negative consequences of this process to prevent distress and to foster well-being. In this way, this paper covers a part of the lacking literature regarding the relation between job crafting and work-family conflict, an element that have to be contemplated, considering the changes in the labour market.

### 1.1. Job Crafting and the JD-R Model

Job crafting has been introduced to describe the process through which employees adapt their job to their preferences and needs. In particular, job crafting refers to the “physical and cognitive changes individuals make in the task or relational boundaries of their work” ([3], p.179). Physical changes concern the amount of job tasks; cognitive changes involve the way the job is seen by the individual; whereas changes in relational boundaries refers to the quality and the quantity of contacts with colleagues [2,9].

Tims et al. [10], extended the concept of job crafting within the theoretical framework of Job Demands-Resources Model (JD-R model [11]), suggesting that job crafting involves the changes made by employees to balance the demands and the resources of their job with their abilities and needs. According to the JD-R Model, Tims et al. [10] identified four job crafting dimensions related to resources and to demands. As for job resources, that are considered aspects of the job that help individuals in achieving goals and in managing job demands [11], Tims and colleagues recognized the dimensions of “increasing structural job resources” (e.g., creating opportunities for professional development and autonomy) and “increasing social job resources” (e.g., active capability in searching support from supervisors and colleagues, or feedback as opportunity of coaching). Within job demands, aspects of the job that require psychological and physical efforts [11], Tims and colleagues identified the dimensions of “increasing challenging job demands” (situations that workers have to overcome to learn and achieve goals) and “decreasing hindering job demands” (requests that impede worker’s personal growth and goal achievement).

Considering that job crafting is particularly characterized by individual’s proactivity [10], this study focuses on the positive dimensions of job crafting, excluding “decreasing hindering job demands”. This is in line with other studies that considered the expansive side of job crafting also within the JD-R model [12,13], linking this process to the possibility to craft both job resources, and challenging demands to reach personal growth, well-being and balance.

This expansive aspect of job crafting, is in line with a meta-analyses conducted by Rudolph et al. [14], suggesting that the overall job crafting and the job crafting dimensions, except “decreasing hindering job demands”, were positively related to the job characteristics, such as job autonomy.

### 1.2. Hypotheses

Within the theoretical framework of the JD-R model, job autonomy is considered a job resource, and it represents the degree of freedom to schedule and manage work. Considering that job crafting is defined as a proactive and also discretionary behavior [3], and that job autonomy can provide the discretionary to activate proactivity and to shape the job, it is expected that job autonomy is an antecedent of job crafting. This is also expected in the light of health dynamics: having the possibility to organize work activity with autonomy, and having the possibility to act this discretionary through job crafting, is a coping strategy with stressful job demands or situations [11].

**Hypothesis** **1.** *Job autonomy is positively associated with job crafting.*

As a job resource, job autonomy is linked to well-being at work and it helps in reducing the perceived distress and experiences characterized by conflict [11]. This study considers work-family conflict, an important construct in the light of the recent changes in the workforce. Work-family conflict is characterized by a process in which the individuals’ behaviours in one domain (work or family) are influenced by the demands and the resources of the other domain [15]. Therefore, conflict is determined by an incompatibility between the work and the family roles [16]. As job autonomy helps managing work, it could support individuals in organizing the management of the work and family domains, reducing the perceived conflict [17]. This is in line with studies suggesting that job resources, such as job autonomy, are positively associated with work-family enrichment, since these resources allow individuals in fitting the work and family domains [7].

Job autonomy can also increase positive experiences and it is linked to well-being indicators at work, such as job satisfaction [11]. Job satisfaction is the result of the evaluation—positive or negative—of the individual on his/her job [18]. Job satisfaction is a crucial construct, from which depend many consequences at the organizational level (i.e., intention to leave the job, absenteeism, individual performance and quality of product/service) and at the individual level (i.e., relationship with life satisfaction, anxiety, distress at work) [19]. Moreover, job autonomy is depicted as antecedent [11], an enhancing element for job satisfaction [4].

**Hypothesis** **2.** *Job autonomy is (2a) negatively associated with work-family conflict and (2b) positively associated with job satisfaction.*

Literature linking job crafting and work-family conflict is very scant. A study by Ilies et al. [20] states that job crafting could be an adaptive response to the perception of work-family conflict, as job crafting can increase the control over the processes influencing work-family conflict, conducting to a balance. Therefore, as job crafting is a proactive behavior contributing to employee well-being [4], it could be reasonable expecting that job crafting may reduce the perception of work-family conflict. Despite this, a recent study by Akkermans and Tims [13] found that job crafting could increase both work-family enrichment, facilitating the management of the two domains, but could also increase work-family interference, as undesirable effect, making boundaries less segmented [21]. Since job crafting is a dimension specifically linked to the job activity and expresses proactive behaviors to adapt job to preferences, motivations, competences, it is possible to suppose that job crafting, focused only on the shaping of the job side and not also on family dynamics, would favor work-family conflict.

On the other hand, literature linking job crafting and job satisfaction is also poor, but empirical evidences show more clearly that job crafting increases employees’ job satisfaction [3,22]. Employees crafting their job are more satisfied, engaged and committed with their organization, and increase their well-being, and this is related to the potential of job crafting. In fact, by shaping job characteristics on skills, abilities and personal preferences, employees make change in the level of job demands and resources, positively influencing their motivation, work engagement and job satisfaction [23].

**Hypothesis** **3.** *Job crafting is (3a) positively associated with work-family conflict and (3b) positively associated with job satisfaction.*

Within the theoretical framework of the JD-R Model, job resources are considered as triggering job crafting [23], it is expected that the hypothesized relation between job autonomy, job crafting, work-family conflict and job satisfaction are confirmed—even stronger—with the mediation of job crafting between job autonomy and work-family conflict, and between job autonomy and job satisfaction.

**Hypothesis** **4.** *Job crafting mediates the association between job autonomy and (4a) work-family conflict and (4b) job satisfaction.*

## 2. Materials and Methods

### 2.1. Sample and Procedure

The sample consists in 389 participants working in a South Italian public administration organization. Starting from about 630 workers employed in the organization, participants are those who decided to participate in the study, with a response rate of 62%. The public administration was selected on the basis of a specific program on work-family balance. This organization, in fact, activated a pilot welfare program on teleworking and, therefore, this organization has been contacted. Unfortunately, when conducting the study, only few workers were involved in the program, therefore it was not possible to introduce this variable. However, the context the context was maintained, also as it was focused on the issues of the study and the quite high response rate is an indicator of this workers’ interest. They are equally distributed within women (50.3%) and men (49.7%), have an average age of 47 years (SD = 8.45), and have a university education or higher (66.8%). Moreover, they have mainly a permanent contract (94.4%), they are employees (87.2%) working full-time (96.5%) for 35 h on average per week (SD = 9.56), and with an average seniority in the organization of 18 years (SD = 9.01).

Data were collected through a self-report questionnaire distributed by researchers, which asked to participants to sign an informed consent. Questionnaires contained a cover letter explaining how to complete the questionnaire, the anonymity and the voluntary nature of participation in the study. Moreover, the letter explicated data processing and privacy according to the code of ethics of the order of psychologists. The organization board of directors gave the permission to conduct the study and participants did not receive any reward. Additional ethical approval was not required because the study did not provide medical treatments or other practices that could origin psychological or social malaises to participants.

### 2.2. Measures

The questionnaire detected the following measures:

Job autonomy was measured on a 7-point Likert scale from 1 (strongly disagree) to 7 (strongly agree) using five items adapted from the Work Design Questionnaire (WDQ) developed by Morgeson and Humphrey [24]. An example item is: “The job allows me to make my own decisions about how to schedule my work”. The reliability coefficient (α) in this study is 0.93.

Job crafting was measured using the Italian version of the scale developed by Tims et al. [10], and adapted by Cenciotti et al. [12], on a 7-point Likert scale from 1 (never) to 7 (always). The Italian version has 13 items and considers three dimensions of job crafting: increasing structural job resources (four items; e.g.,: “I try to develop my capabilities”; α = 0.78), increasing social job resources (four items; e.g.,: “I ask others for feedback on my job performance”; α = 0.71), and increasing challenging demands (five items, e.g.,: “When an interesting project comes along, I offer myself proactively as project co-worker”; α = 0.76). The overall 13-item scale has α = 0.83.

Work-family conflict was measured using the Italian adaptation of the scale developed by Netemeyer et al. [25], adapted by Colombo and Ghislieri [26]. The scale is composed by five items, on a 6-point Likert scale from 1 (never) to 6 (always), an example item is: “The demands of my work interfere with my home and family life”, α in this study 0.90. Job satisfaction was measured using 5 items by Judge, Locke, Durham, and Kluger [27], on a 7-point Likert scale from 1 (strongly disagree) to 7 (strongly agree). An example item is: “I feel fairly well satisfied with my present job”, α in this study 0.72.

### 2.3. Data Analyses

Descriptive statistics, alpha reliabilities (α) for each scale, and correlations (Pearson’s r) between variables were performed with SPSS 24 (IBM, Armonk, NY, USA). To examine the potential effects of common method bias, three different models were compared: (M1) the hypothesized four-factor model (job autonomy, job crafting, work-family conflict, job satisfaction), (M2) a model with a single factor, and (M3) a model in which the items loaded both on their expected factor and on a latent common method factor [28]. 

Furthermore, to investigate the divergent validity of the constructs, (M1) was also compared to three-factor model where job autonomy and job crafting were replaced by a single “predictors” (M4).

In order to test the mediating role of job crafting between job autonomy and work-family conflict and job satisfaction, a structural equations model was estimated with MPLUS 8. Hypotheses were specified a priori and it was performed a partial mediation model [29]. 

Goodness of fit of the model was evaluated by the chi-square value (*χ*^2^), the Comparative Fit Index (CFI), the Tucker Lewis Index (TLI), the Root Mean Square Error of Approximation (RMSEA), and the Standardized Root Mean Square Residual (SRMR).

Latent variables are composed by indicators built by the parceling method and each latent variable was composed by two or three parcels (indicators composed by two or more items on average). The parceling method is more advisable since it reduces type I errors in item correlations, and it lessens the likelihood of a priori model misspecification [30,31].

According to the a priori specification model and to the Italian validation of the job crafting scale [12] used in this study, which showed a robust correlation between the two dimensions of increasing structural job resources and increasing challenging demands, a correlation between these two parcels was added in the estimated model. Moreover, to detect possible differences in the perception of the explored variables, it was performed an analysis of variance.

## 3. Results

Firstly, the comparisons between the alternative models (Table 1) suggested that the hypothesized model M1 fitted data better than the one-factor model M2. The fit of M1 was not superior to the fit of M3 which explained the 5.8% of the variance. This is below the average of 25% found by Williams, Cote and Buckely [32], suggesting that common method variance does not significantly influence results. Furthermore, M1 significantly fitted the data better than M4. In sum, M1 was used to test the study hypotheses [28].

From a psychometric standpoint, all variables assessed in the study show satisfactory Cronbach’s alphas ranging between 0.72 and 0.93. Moreover, average scores of each variable exceed the central point of scales (Table 2), remarking the existence of these constructs among this population.

As for correlations, job crafting shows significant and positive correlations with all variables, in particular with job satisfaction (*r* = 0.38), with job autonomy (*r* = 0.33), and a weak relation with work-family conflict (*r* = 0.16) which, in turn, shows a higher negative correlation with job satisfaction (*r* = −0.27). The estimated structural equations model shows satisfactory fit indices, which confirms the goodness of the model fit: *χ*^2^ (20) = 33.008, *p* < 0.05, CFI = 0.99, TLI = 0.98, RMSEA = 0.04, SRMR = 0.03. Moreover, the structural equations model shows parcels with significant loadings (*p* < 0.001). 

**Figure 1:**
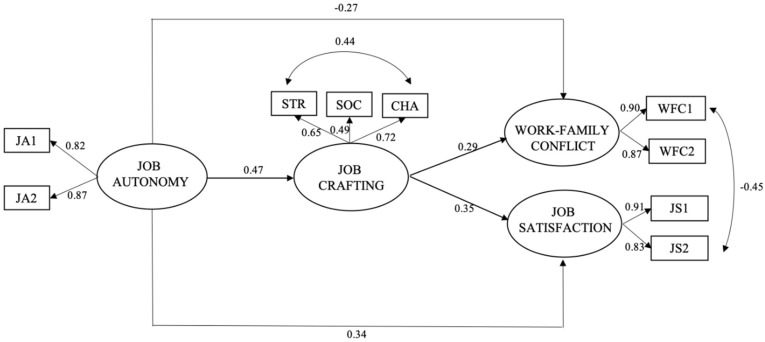
Results of the structural equations model. Note. STR = increasing structural job resources; SOC = increasing social job resources; CHA = increasing challenging demands; JA1 = parcel 1 of the latent variable job autonomy; JA2 = parcel 2 of the latent variable job autonomy; WFC1 = parcel 1 of the latent variable work-family conflict; WFC2 = parcel 2 of the latent variable work-family conflict; JS1 = parcel 1 of the latent variable job satisfaction; JS2 = parcel 2 of the latent variable job satisfaction.

Deepening the model (Figure 1), job autonomy is directly associated with all variables, in particular positively with job crafting (β = 0.47), and with job satisfaction (β = 0.34), and negatively with work-family conflict (β = −0.27), thus confirming hypothesis 1 and hypothesis 2a and 2b. Job crafting shows positive and significant associations with both job satisfaction (β = 0.35), and work-family conflict (β = 0.29), confirming hypotheses 3a and 3b.

Moreover, in this study, job crafting confirms its mediating role since the model shows positive significant and indirect associations between job autonomy and job satisfaction (β = 0.16), and between job autonomy and work-family conflict (β = 0.13) through job crafting (Table 3), confirming hypotheses 4a and 4b. 

The correlation between two parcels of job crafting, increasing structural job resources and increasing challenging demands, did not modify fit indices and increases the level of mediation, showing an association between two dimensions linked to engagement and development to reach job tasks. Indeed, as postulated a priori, this result is supported and confirmed also by the psychometric characteristics of the scale, since the Italian validation of job crafting scale [12] shows a strong correlation between these two dimensions (*r* = 0.92), highlighting the coherence from both theoretical and methodological standpoints. Moreover, the significance of the hypothesized mediation was confirmed by the bootstrapping procedure with CI not including zero, confirming also the partial mediation expected a priori. Finally, it has to be noted that analysis of variance did not show significant differences between male and female in the experimentation of variables included in the study.

## 4. Discussion

This study focused on job crafting as a mediator, to detect and understand its relation with one positive outcome, job satisfaction, and one negative outcome, work-family conflict. Moreover, the study considers job autonomy as antecedent of job crafting and other outcomes, operationalizing it as a job resource within the theoretical framework of the JD-R model. 

This study significantly contributes to the job crafting literature, which lacks in detected relations with negative outcomes, in particular work-family conflict. Hypothesis 1, which stated job autonomy as antecedent of job crafting, confirmed this relation. Job autonomy is important for employee well-being and for the possibility to cope with workload and job demands, protecting from stressful situations. Beyond the JD-R model, other studies found that job autonomy can activate positive experiences at work [33], preventing distress and negative outcomes, and favoring positive dynamics linked to the optimal level of job challenge to manage [34]. Moreover, job autonomy is depicted as antecedent of job crafting identifying resources as opportunities for crafting employees’ job [14,35]. Referring to the confirmed hypotheses 2a and 2b, job autonomy as a resource, can protect employees from distress, and activates positive and motivating processes leading to well-being and satisfaction [11], allowing employee to manage work and personal spheres [7]. Indeed, in this study, having autonomy, discretionary over the job and the organization of different activity, is a crucial resource to reduce the perception of conflict between the demands of the work and family domains, and to cope with the many daily demands [36]. 

Hypothesis 3 shows interesting and remarkable findings, which particularly contribute to the literature linking job crafting and work-family conflict. Considering the potential of job crafting in increasing the control over work process, its proactivity characteristic and its focus on the specific job activity, it was expected that job crafting had a positive relation with work-family conflict. This study showed, in fact, a positive relation between job crafting and work-family conflict, confirming hypothesis 3a, and revealing a new noteworthy aspect of job crafting, which seems to foster a negative outcome. As for the many changes in the workforce and the labour market with new job design also characterized by job crafting, many organizations are now searching for people showing effort, initiative and proactivity [37]. This highly demanding framework, could lead workers to proactive behaviours and job crafting processes, leading them, on the other hand, in working more than in other circumstance. This situation, in fact, could lead to dysfunctional behaviours, making employees working without boundaries and times, due to new working processes, but also due to new technologies that allow that work issue reach people also in the family or private life domains. This may involve difficulty in manage the demands coming from the family domain, causing the perception of work-family conflict. This is in line with a study by Akkermans and Tims [13] which states that expansive job crafting behaviours could raise work-home interference. Results related to work-family domains and covering the expansive job crafting aspect, are congruent with the meta-analysis by Rudolph et al. [14], in which job crafting was negatively correlated with job strain and emerged that the dimensions of increasing job resources, both structural and social, may function to offset the negative influence. Since in the present study job crafting is declined in its expansive dimension, not considering the dimension of “hindering job demands”, it could be that this also contributed to the positive relation found between job crafting and work-family conflict. This opens important considerations for literature and for the applicability of job crafting that should be deepen in future studies.

Hypothesis 3b, which expected the positive relation between job crafting and job satisfaction, is confirmed. Job crafting acts its positive effect on motivation and well-being dynamics, highlighting the positive result of modelling skills, abilities and preferences, as job crafting allows. In line with the choice to consider the expansive side of job crafting, a study by Petrou et al. [38] found that job satisfaction was associated to resources, and also to the challenging elements of job crafting. This particular association is also in line with the relation added in the structural equations: the correlation between the increasing structural job resources parcel with the increasing challenging demands parcel, empowered the model from an empirical and methodological standpoint, but also confirmed job crafting as an element linked to stimulating, motivating and also satisfactory jobs and environments. 

As for hypothesis 4, findings follow the trend of hypothesis 3. In fact, the relation between job autonomy and work-family conflict through the mediation of job crafting is positive, confirming hypothesis 4a. This is interesting because if job resources enhance job crafting [23], it is desirable that job autonomy strengthen job crafting which, in turn, confirms its effect on work-family conflict. This is another crucial result of this study, since it highlights a new element of job crafting as a mediator. The interaction between job crafting as a mediator and the antecedent of job autonomy, could be read in the light of its relation with job resources and their capacity in empowering each other: job crafting is a key to decode resources in well-being and balance [13], but job autonomy can make indefinite the boundaries between work and family (e.g., by carrying on work at home, and extending working hours at home), fostering sliding from a domain to the other [39]. Therefore, also job crafting could lead to loss of control, in a perspective of a vicious circle with a job resource. 

Hypothesis 4b is confirmed. Job autonomy has a positive relation with job satisfaction through the mediation of job crafting, suggesting the mutual contribution between job autonomy and job crafting. This highlights job crafting as mediator in well-being dynamics, and job autonomy as antecedent on job crafting within their common elements of discretionary and proactivity. 

Finally, the analysis of variance did not show, in this study, any significant differences between male and female. This result could be read in the light of the pilot welfare program on teleworking and, therefore, on the sensitization on the issue of work-family conflict, directly involving the themes of job crafting and job autonomy.

### Limitations and Future Studies

A limitation of this study is the sample, involving only one context, making findings not representative of employees and organizations. Despite this, a study by Johns [40] suggests that a context could have powerful effect and can be a strength element, responsible of different impacts on the detected variables, giving stability since variables become constants among a specific context, also at the cross-level effect. According to Johns [40], the used context can be reinterpreted in a positive framework: it might work as a variable interacting with personal factors to influence organizational behaviour. In the organization participating to this research, many welfare actions have been started. Considering the current social scenario (older workers, people ageing), the activated welfare programs (smart working, part-time jobs) reflect the current social and personal needs. Employees receive support through welfare services, improving their job crafting behaviours and, thus, finding new strategies to reduce negative outcomes such as work-family conflict. Moreover, the use of these welfare actions or the sensitization on the topics, could be an other explanation of the non significance of the analysis of variance resulted in this study.

However, to capture diversities and multiple characteristics among professions and organizations, future studies should consider multi-group analysis to suggest focused practical implications. Moreover, as this study involved an organization with mostly highly educated participants, future studies should also consider the effect of lower education levels. This could be functional to detect if job autonomy could have the same role in activating job crafting behaviours or if it could be better a guide or more support, in particular to learn and gain awareness on how crafting one’s own job.

This study used a cross-sectional design that does not permit to establish definite causality relationships between variables. Longitudinal studies should detect job crafting on a daily basis, to understand if job satisfaction and other constructs related to well-being depends on the particular experience of job, or depends by the long term experience, as suggested by Plomp et al. [41]. This could be useful to detect the relation between job crafting and work-family conflict, also in the daily management of work and home domains. Finally, future studies should detect job crafting through behaviours oriented to proactively adapt job to personal or family needs, to focus job crafting on this specific dynamic which links job and the individual.

## 5. Conclusions

This study contributes to literature by confirming the importance of job crafting for employees’ positive outcomes, like job satisfaction. Moreover, this study highlights an important dynamic played by job crafting which, also linked to the discretionary of job autonomy, can rise negative outcomes, such as work-family interference. This study considered new frameworks of the labor market concerning new job processes and new compositions of the workforce, and emphasized a negative aspect of job crafting. Indeed, a previous study by Oldham and Hackman [42] identified the possible dysfunctional consequences of job crafting: employees can adapt job to their preferences, skills and needs, but this particular job design may lead to unwanted consequences [23] both for the organizations and the person. In this study, it could be read in the light of crafters who may manage their work raising negative spillover effects as work-family conflict. 

Therefore, human resources managers should consider implications related to job crafting processes. In the light of beneficial consequences of job crafting on employees, such as better performance and productivity, organizations should offer specific training programs [43]. These are functional both to understand what happens when job crafting is activated, and to recognize the possible consequences of a wrong self-organization. Employees may have the possibility to try to craft their job and to correct it if not productive or well-being oriented. An example is the case of job crafting which may be used to decrease tasks, decreasing also performance [44], or the present study that highlights the possibility to experiment work-family conflict. It is therefore important to create awareness within managers and employees to concretely take advantage from job crafting. Making employees aware about job processes, is important also considering job autonomy as a resource, but if lacks awareness, it could be translated into over work and work beyond the working time, leading to work-family conflict, but also to workaholism [39] with consequences on health [45]. Moreover, it is important to offer instruments to face the work-family conflict: is it possible to consider job crafting a sort of family-friendly strategy? If the training project is adequate, job crafting could be a strong instrument to reduce work-home interference and job autonomy could reinforce this strategy. Having autonomy, in fact, make employees able to recognize priorities, tasks and goals, leading them to better craft their job and, consequently, to experiment motivation and well-being. In this sense, organizations should monitor the available job resources and the level of job demands. This study focused on the expansive job crafting, but from an applicative standpoint it could be important to detect and make possible that employee meeting also hindrance job demands receive adequate job resources. In the light of resources, collaborative job crafting [44], implying that employees design together their job, may activate a sort of supportive organizational context enhancing satisfaction and well-being, since supportive colleagues and supervisors are crucial resources in reducing work-family conflict [21].

## Figures and Tables

**Table 1:** Fit indices and models comparisons.

Measurement Model	χ2	df	*χ*^2^/df	CFI	NNFI	RMSEA	SRMR	Model Comparison	Δ*χ*^2^	df	*p*
M1 measurement model	1537.76	492	3.12	0.853	0.812	0.074	0.054				
M2 one-factor model	6182.35	594	10.41	0.217	0.169	0.156	0.157	M2-M1	4644.59	102	<.001
M3 common factor model	1273.45	460	2.77	0.886	0.844	0.067	0.041	M1-M3	264.31	32	<.001
M4 three-factor model	3282.29	591	5.55	0.627	0.602	0.108	0.113	M4-M1	1744.53	99	<.001

Note. CFI: comparative fit index,NNFI: non normed fit index,RMSEA: root mean square error of approximation, SRMR standardized root mean square residual

**Table 2:** Means, Standard Deviations and Correlations—Pearson’s *r*.

Variables	M	SD	1	2	3	4
1. Job Crafting	4.95	0.87	(0.83)			
2. Job Autonomy	4.88	1.44	0.33 **	(0.93)		
3. Work-Family Conflict	3.39	1.32	0.16 *	−0.12 *	(0.90)	
4. Job Satisfaction	4.65	1.27	0.38 **	0.42**	−0.27 **	(0.72)

Note. ** *p* < 0.01 level; * *p* < 0.05 level. Cronbach’s alpha’s on the diagonal (between brackets).

**Table 3:** Indirect effects of the estimated SEM using bootstrapping.

Indirect Effects	Standardized Indirect Effects—Bootstrapping Procedure			
	Est.	s.e.	*p*	CI 95%
Job autonomy → Job crafting → Job satisfaction	0.16	0.03	0.01	(0.08, 0.40)
Job autonomy → Job crafting → Work-family conflict	0.13	0.04	0.01	(0.06, 0.39)

Note. Est = estimated; s.e. = standard error; CI = confidence interval.
